# Compressive Mechanical Properties of Porcine Brain: Experimentation and Modeling of the Tissue Hydration Effects

**DOI:** 10.3390/bioengineering6020040

**Published:** 2019-05-07

**Authors:** Raj K. Prabhu, Mark T. Begonia, Wilburn R. Whittington, Michael A. Murphy, Yuxiong Mao, Jun Liao, Lakiesha N. Williams, Mark F. Horstemeyer, Jianping Sheng

**Affiliations:** 1 Center for Advanced Vehicular Systems, Mississippi State University, Mississippi State, MS 39795, USA; mbegonia@vt.edu (M.T.B.); whittington@me.msstate.edu (W.R.W.); mam526@cavs.msstate.edu (M.A.M.); maxmcn@gmail.com (Y.M.); 2 Department of Agricultural & Biological Engineering, Mississippi State University, Mississippi State, MS 39762, USA; 3 Department of Mechanical Engineering, Mississippi State University, Mississippi State, MS 39762, USA; 4 Department of Bioengineering, University of Texas Arlington, Arlington, TX 76010, USA; jun.liao@uta.edu; 5 J. Crayton Pruitt Family Department of Biomedical Engineering, University of Florida, Gainesville, FL 32611, USA; lwilliams@bme.ufl.edu; 6 School of Engineering, Liberty University, Lynchburg, VA 24515, USA; mhorstemeyer@liberty.edu; 7 U.S. Army Tank Automotive Research, Development, and Engineering Center (TARDEC), Warren, MI 48397, USA; jianping.sheng.civ@mail.mil

**Keywords:** porcine brain, mechanical behavior, hydration effects, Split-Hopkinson pressure bar, micromechanics, finite element analysis

## Abstract

Designing protective systems for the human head—and, hence, the brain—requires understanding the brain’s microstructural response to mechanical insults. We present the behavior of wet and dry porcine brain undergoing quasi-static and high strain rate mechanical deformations to unravel the effect of hydration on the brain’s biomechanics. Here, native ‘wet’ brain samples contained ~80% (mass/mass) water content and ‘dry’ brain samples contained ~0% (mass/mass) water content. First, the wet brain incurred a large initial peak stress that was not exhibited by the dry brain. Second, stress levels for the dry brain were greater than the wet brain. Third, the dry brain stress–strain behavior was characteristic of ductile materials with a yield point and work hardening; however, the wet brain showed a typical concave inflection that is often manifested by polymers. Finally, finite element analysis (FEA) of the brain’s high strain rate response for samples with various proportions of water and dry brain showed that water played a major role in the initial hardening trend. Therefore, hydration level plays a key role in brain tissue micromechanics, and the incorporation of this hydration effect on the brain’s mechanical response in simulated injury scenarios or virtual human-centric protective headgear design is essential.

## 1. Introduction

Traumatic brain injury (TBI), due to mechanical impact to the head, is a leading cause of death and life-long disability in the United States. Around 5.3 million Americans currently have long-term disabilities after sustaining a TBI [1]. In the United States, direct and indirect medical costs related to TBI amounted to an estimated $60 billion for the year 2000 alone [2]. Thus, TBI’s profound impact on our society necessitates effective protective measures to curb consequent injuries and disabilities [3]. Ostensibly, to design protective equipment for the brain requires an understanding of its mechanical response during injurious loading conditions. Thus, microstructural investigation of the relationship between tissue hydration, cellular structure, and mechanical impact at various strain rates is critical to effective comprehension and modeling of underlying TBI mechanisms.

In the 1940s, pioneering work on the mechanical properties of brain parenchyma asserted that shear strain had a significant influence on brain trauma during an impact or at finite deformations [4,5]. Motivated by the shear strain theory, a number of subsequent studies on brain mechanical properties concentrated on shear experiments [6] and quantifying shear properties [7,8,9,10]. The strain rates for the experiments ranged from quasi-static to moderate strain rates (0.001–60 s^−1^) and revealed a nonlinear stress–strain behavior. Hence, the brain was treated as a soft engineering material. A seminal study by Estes and McElhaney [11] on brain specimens under quasi-static compression showed a similar nonlinear response (with stiffening stress–strain behavior). In more recent studies, extensive shear tests were performed on human brains in the strain rate range of 0.1–90 s^−1^ [12]. Their results confirmed the earlier nonlinear stress–strain behavior.

Motivation to understand the role of axonal fibers (brain white matter) spurred a series of shear relaxation experiments on brain and brain stem materials, which showed strain-rate dependent behavior at quasi-static rates [13]. Furthermore, their studies noted the anisotropic nature of a brain due to the presence of axonal fibers, mainly in the quasi-static regime with the white matter being stiffer than grey matter [14]. White matter’s greater stiffness was attributed to the fibrous texture of axons. Essentially, the difference in stiffness of white and grey matter contributed to the variation of stress–strain behaviors in the cerebrum, cerebellum, and brain stem especially at strains larger than 20% [15,16,17].

Similar nonlinear (hardening) mechanical behavior was also observed in tensile studies conducted at the turn of the 21th century [18,19]. The strain rates that were employed were in the quasi-static regime showing a high strain rate sensitivity. In another tensile response study, in-vitro testing on a cultured human brain showed subsequent swelling of neurons similar to that observed in the rat’s brains after a TBI [20]. The axonal fibers demonstrated a delayed elastic response after an initial dynamic stretch injury. The evolution of this elastic response involved immediate undulations of axonal fibers after the dynamic stretch injury, which was followed by a slow reversion to its original shape (straight orientation) within an hour [21]. 

More recently, moderately-high-strain-rate compression tests showed a minor dependence on the heterogeneity of brain for strain rates greater than 40 s^−1^ [22]. Other noteworthy research established the premise for the onset of TBI in in-vitro brain tissue cultures in strains larger than 20% at strain rates greater than 40 s^−1^ [20,23,24]. Consequently, in the current study, strain rates greater than 50 s^−1^ were treated as high rate.

Initial hardening from the high strain rate behavior of soft biological materials was not evident in the quasi-static behavior where the stress monotonically increased as the deformation proceeded [18,19,22,25]. However, moderately high strain rate data (10–70 s^−1^) of the human liver tissue were predominantly marked with an initial hardening trend that was followed by a softening and then further hardening at larger strains [26]. Some studies argued that the initial hardening was due to inertial effects and asserted that an annular geometry of the specimen averted the inertial effects and guaranteed a uniform stress-state [27,28]. However, in a recent study on the dynamic response of the brain, part of the inertial effects was shown to be intrinsic to the material and that non-uniform stress-states were realized in the material [29]. As a major component of the brain, water proved crucial in instigating the initial hardening [29,30]. To date, no researcher has analyzed the effect of the amount of water within the brain that has undergone high impact loads. Cheng and Bilston [31] did show that the quasi-static viscoelastic properties of brain white matter arose from the solid matrix with the interstitial fluid’s migration in the white matter, providing the short-term elastic response, and modeled the white matter’s response using a poroviscoelastic (PVE) model. However, the work of Cheng and Bilston [31] did not directly address the hydration effects at higher strain rates.

The contribution of our current study is the quantification of the difference in the mechanical properties of the wet and dry brain at quasi-static and high strain rates. We then present the micromechanics of brain’s high rate deformation, based on a mixture theory of water and dehydrated brain, through finite element (FE) simulations. Section 2.1 and Section 2.2 describe the materials and methods employed in the experiments. Section 2.3 describes the theory employed in the experiments, and Section 2.4 presents the statistical methodology employed for experimental data. Section 2.5 presents the theory for micromechanics and gives an overview of the Split-Hopkinson pressure bar (SHPB) FE simulations used to evaluate the micromechanics of water included in the brain and the dry brain in the specimen. Section 3 presents the results and the corresponding discussion. Finally, conclusions are summarized in Section 4.

## 2. Materials and Methods

The experimental procedures involved in this study used in vitro porcine samples, the protocol for which were approved by the Office of Regulatory Compliance and Safety (ORSC) at Mississippi State University (MSU), Mississippi State, MS, 39762. The IACUC approval number is 11-048.

### 2.1. Sample Preparation

Intact porcine heads from healthy males were collected from a local abattoir and transported to a necropsy laboratory (Mississippi State University College of Veterinary Medicine). Porcine brains were extracted from each skull and stored in a phosphate buffered saline (PBS) solution to minimize dehydration and degradation. Test specimens were prepared via scalpel incision through the corpus callosum to separate the two hemispheres. A stainless-steel cylindrical die was then used to dissect brain material through the sagittal plane of each hemisphere, producing cylindrical test specimens with the sulci and gyri characterizing the superior surface. Each test specimen consisted of gray and white matter. The average initial height of the test specimens was 15 mm while the average initial diameter was 30 mm. Brain extractions and dissection required approximately one hour for completion, and all compression experiments were conducted within three hours post-mortem [25]. For making ‘dry brain’ specimens, surgically extracted cylindrical porcine brain samples were lyophilized using a Freezone™ 1-liter benchtop freeze dryer (LABCONCO ^®^) for approximately 48 hours under 0.1 Pa and −50 °C. The lyophilizing process evaporated the water in the brain, giving the lyophilized parenchyma. Before testing, a 30 mm diameter die was used to dissect the lyophilized tissue specimens with an average thickness of 15 mm. Details of the samples obtained and porcine brains used along with testing conditions are given in Table 1.

### 2.2. Testing Apparatuses

#### 2.2.1. Mach-1™ for Quasi-static Testing of the Wet Brain

**Figure 1:**
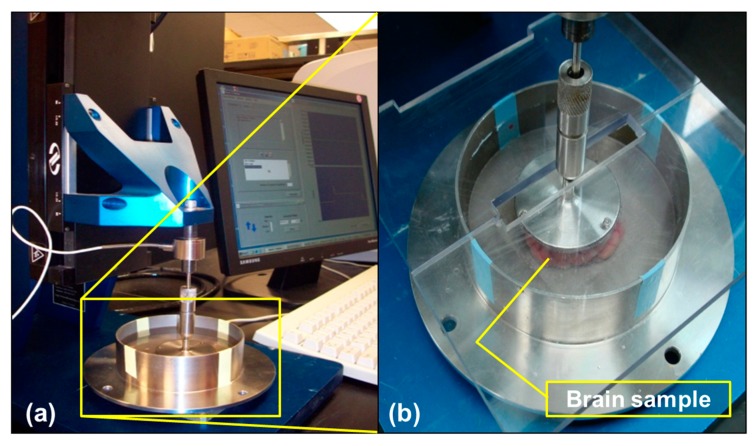
(**a**) Quasi-static compression test on the porcine brain using the Mach-1™ Micromechanical Testing System (BIOMOMENTUM, Quebec). (**b**) Wet porcine samples were immersed in 0.1 M neutral buffered PBS during quasi-static compression at strain rates of 0.00625, 0.025, and 0.1 s^−1^.

The MACH-1™ Micromechanical Testing System (BIOMOMENTUM, Quebec), Universal Motion Controller/Driver—Model ESP300 and load cell amplifier were used for the quasi-static compression experiments (Figure 1). The 1 kg (10 N) load cell had a resolution of 0.50 mg and was included with the Mach-1™ Micromechanical Testing System (BIOMOMENTUM, Quebec) to meet the sensitivity requirements for testing porcine brain tissue (~80% water content, referred to as wet brain) [32]. A circular platen with an estimated diameter of 50 mm was also selected to accommodate the smaller cross-sectional area of the test specimens [25]. In addition, a stainless-steel chamber was fabricated for housing test specimens immersed in 0.01 M PBS, prior to testing to minimize tissue dehydration. The samples were tested to their failure strains at applied strain rates of 0.00625, 0.025, and 0.1 s^−1^.

#### 2.2.2. Split-Hopkinson Pressure Bar (SHPB) for High Strain Rate Testing of Wet and Dry Brain Specimens

**Figure 2:**
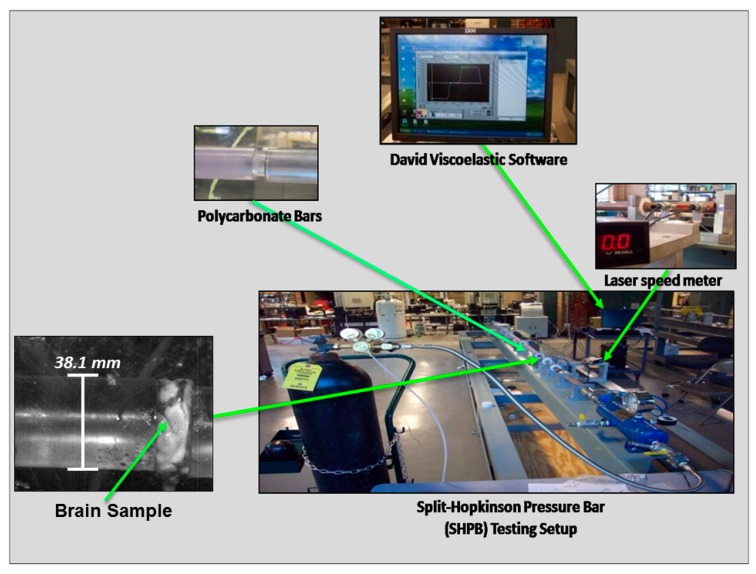
Overview of the Split-Hopkinson pressure bar (SHPB) used to conduct high-strain-rate tests on porcine brain specimens. Strain rates ranged from 50 to 750 s^−1^.

The Split-Hopkinson pressure bar (SHPB) comprises a striker bar, an incident bar, and a transmitted bar (Figure 2) [33,34,35] (see Appendix B). For both wet and dry brain, each specimen (30 mm diameter, 15 mm thickness) was placed between the incident and transmitted bars. The striker bar was then propelled at a specified velocity via a pneumatic device, hitting the incident bar and causing compression on the specimen lodged between the two aforementioned bars, which were instrumented with strain gauges to collect the corresponding data. The experimental setup consisted of DAQ modules, strain gauges, a laser speed meter, a pressure release valve, and polycarbonate bars (Figure 2). The strain gauge data was processed using David Viscoelastic software [29,36,37]. Using the SHPB apparatus, wet brain specimens were tested at 50, 250, 450, 550, and 750 s^−1^, and dry brain specimens were tested at 250 s^−1^.

#### 2.2.3. Instron™5568 for Quasi-Static Testing of the Dry Brain

**Figure 3:**
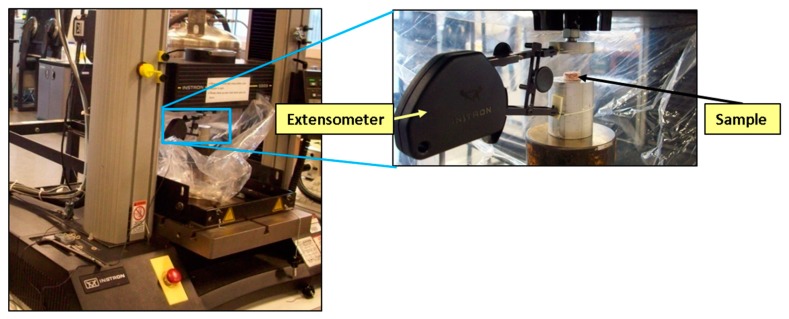
Instron™ 5869 configuration used for compression testing of lyophilized (dry) porcine brain samples at strain rates ranging from 0.00625 to 0.1 s^−1^.

**Figure 4:**
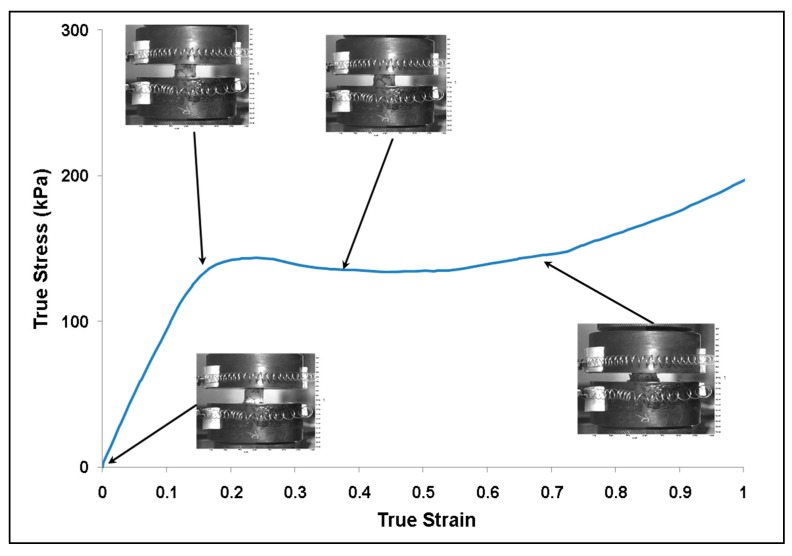
Plot of true stress–strain behavior for the lyophilized (dry) brain at a strain rate of 0.1 s^­−1^. The images show the deformation of the specimen during testing. The specimen started to barrel above 40% true stain. The water content in the specimen was 0% m/m.

Quasi-static rate compression tests on the lyophilized porcine brain were performed using an Instron™ 5869 load frame (Figure 3). Due to the stiffer nature of the lyophilized dry brain, a larger load cell capacity (1 kN) was needed when compared to wet brain testing, and hence an Instron™ 5869 was used instead of the Mach-1™. Lyophilized brain specimens were cylindrical in geometry with a diameter of 15 mm (1 mm tolerance) and a height of 8 mm (2 mm tolerance), which was measured using digital calipers (Mitutoyo 500-752-10 CD-6” PSX). The strain was recorded using an Instron™ mechanical extensometer, which was used to control the strain rates of the experiments. Post-processing of the data was performed using Bluehill software (Instron™) at strain rates of 0.00625, 0.025, and 0.1 s^−1^. Video imaging using LaVision™ software was also recorded on tests at strain rates of 0.00625, 0.025, and 0.1 s^−1^ to investigate the onset of barreling in samples and found that no barreling was observed up to 40% true strain (Figure 4).

### 2.3. Stress–Strain Experimental Data

Determining the true stress–strain behavior for the porcine brain at quasi-static rates were performed using standard procedures and formulations [38]. While testing wet and dry brain samples, using the Mach-I and Instron™ 5869 machines, respectively; precautions were taken to ensure a uniform stress state during testing. The LaVision™ video/software suite was used to ensure that the data measured were under a uniform cross-sectional area of the specimen and hence uniform stress state. For high strain rate tests using the SHPB, stress–strain calculations were made using standard wave theory equations. A detailed discussion on wave theory and its application to the SHPB is presented by Gary et al. [36] and Prabhu et al. [29,37].

During analysis, the tangent modulus was calculated based on the slope of the stress–strain response at 5% strain, the elastic–inelastic transition stress were determined based on the yield stress at high strain rates, and the quasi-static wet brain data was asserted to have elastic–inelastic behavior subsequent to 5% strain.

### 2.4. Statistical Analysis of the Experimental Data

Statistical analyses of three parameters, namely the tangent modulus, elastic–inelastic transition stress (σ_p_ or σ_t_), and the true strain at σ_p_ or σ_t_ at various strain rates were conducted using the SigmaStat 3.0 software (SPSS, Chicago, IL). A one-way analysis of variance (ANOVA) method was used for statistical analysis on the three parameters, with a Holm-Sidak test being used for post-hoc comparisons. The mechanical difference at various strain rates, for a particular parameter, was considered to be statistically significant when *p* < 0.05.

### 2.5. Finite Element Simulation-Based Micromechanics of the Dry Brain and Water

**Figure 5:**
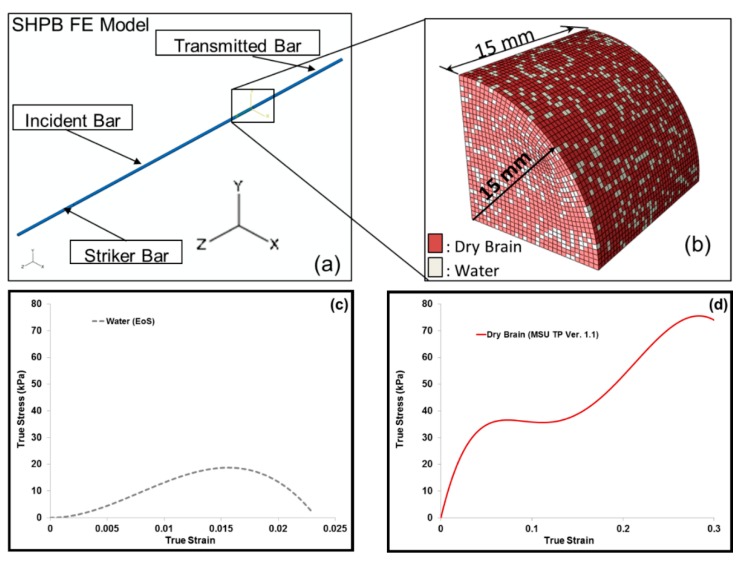
(**a**) A schematic of the finite element (FE) set up for Split-Hopkinson pressure bar (SHPB) tests and (**b**) FE simulation sample dimensions, with a sample having 20% (m/m) water and 80% (m/m) dry brain. The water content in the dry brain is ~0% (m/m). Local loading direction (negative z-direction) true stress–strain responses of (**c**) water and (**d**) dry brain. Mixture theory was applied to obtain the entire sample’s mechanical response.

FE simulations of the SHPB setup were similar to the experimental SHPB setup (Figure 5a). An FE simulation was initialized by allowing the striker bar to be set in motion. The speed of the striker corresponded to that of the SHPB experiment. As the striker bar came into contact with the incident bar, stress waves that arose from the striker bar propagated through the incident bar. Part of the wave traveled through the sample and part was reflected back into the incident bar. The applied striker bar speed and the resulting pressure became the boundary conditions for the FE simulations of the whole SHPB set-up with a reduced integration formulation and default hourglass controls. In all, the number of elements included in the SHPB FE model was 47,300, and the type of elements used were regular hexahedrons. The striker, incident and transmitted bars were treated as elastic materials. The Young’s Modulus and Poisson’s ratio were 2391.2 MPa and 0.36, respectively. Figure 5a gives an overview of the FE model that simulated the SHPB test. The FE simulation sample elements were randomly assigned materials properties of water and dry brain (Figure 5b) such that the effective mass of the water and dry brain elements in the specimen varied from 20% to 80% m/m. Table 2 gives the FE simulation cases from the combination of water and dry brain. Here, a micromechanics approach of calculating the total average stress of the specimen from the component level stresses, that is, of water and the dry brain was implemented. 

**Figure 6:**
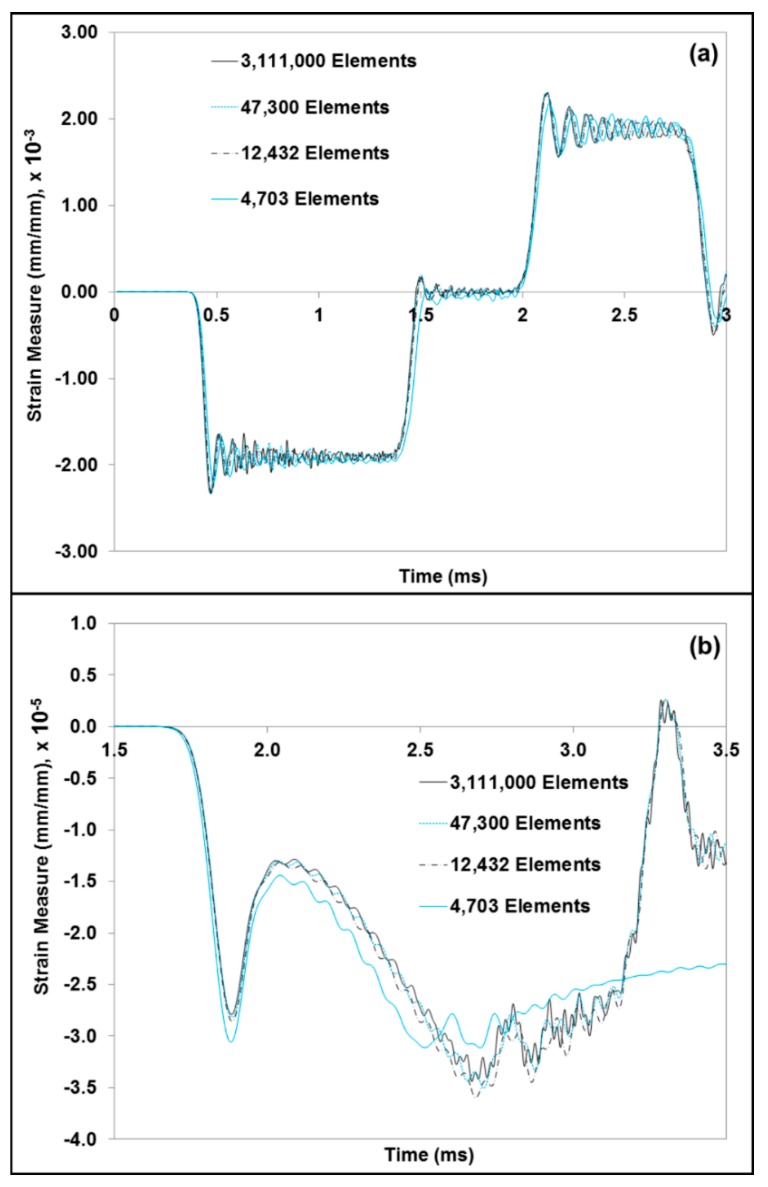
Comparison plots from the FEA mesh refinement study, which shows the strain measures for the (**a**) incident and reflected wave and (**b**) transmitted wave. The element type considered for this study was regular hexahedral. Four cases of mesh refinement, varying from 4703 to 3,111,000 elements, were considered.

**Figure A2:**
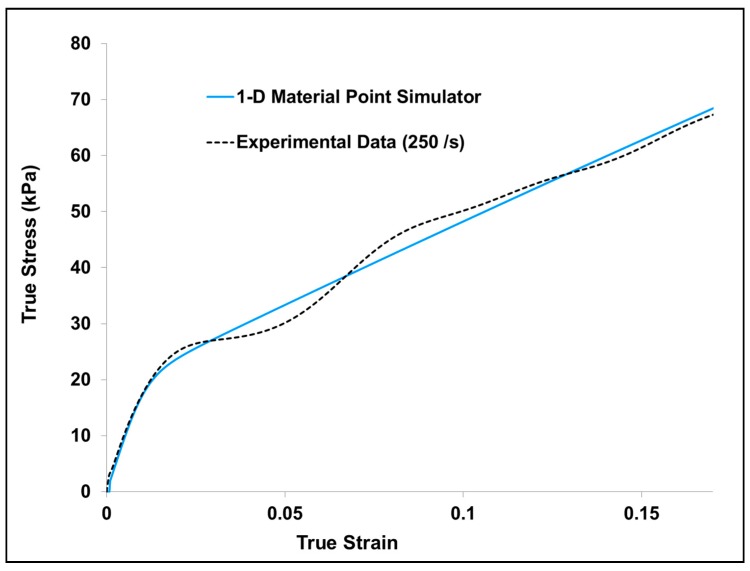
Comparison of experiment and one-dimensional (1-D) material point simulator σ_33_ for dry porcine brain sample compression at 250 s^−1^. The material parameters for MSU TP Ver.1.1 material model were calibrated to the experimental data.

An Equation of State (EOS) was used to define the material property of water. The EOS assumed the Hugoniot form of Mie-Gruneisen’s EOS [39]. The expression for the EOS used for water in the FE model is as follows in Equation (1)

(1) $$U_{s}=c_{0}+sU_{p}$$

where $U_{s}$ is the wave velocity, $U_{p}$ the particle velocity and $c_{0}$ and *s* are constants of the linear relationship of the wave velocity and particle velocity. A specific material model, MSU TP 1.1 [40], was used to capture the elastic and inelastic response of the dry brain material. A summary of the constitutive equations is shown in Table 3 (see Appendix A for additional details). The constitutive model (MSU TP Ver. 1.1) presented in this paper captures both the instantaneous and longer-term large deformation processes and could admit microstructural features within the internal state variables. With the microstructural features, we can use our internal state variable model so that eventually history effects could be captured and predicted. In the absence of the microstructural features, other constitutive models should be able to show the stress state under the high-rate loadings exhibited here since no varying history was induced. A one-dimensional version of the material model, MSU TP Ver. 1.1, was implemented in MATLAB [41] to obtain the material point simulator. The material point simulator was then optimized to calibrate the material model constants to the high strain rate experimental data of the dry brain at 250 s^−1^ (Figure A2). The values for the material constants for MSU TP 1.1 are shown in Table 4, Figure 5c,d give the local representative mechanical responses of water and dry brain, respectively. They denote the local elemental response for the FE model specimen consisting of a mixture of water and dry brain. Initially, a mesh refinement study for the FE model was also conducted to analyze the convergence of ABAQUS/Explicit solutions [42]. Figure 6. shows the simulation results of the incident and transmitted strain measurements at different mesh resolutions, respectively. Figure 6. also illustrates that meshes with 4703 and 12,432 elements did not converge, but the FE solutions with 47,300 elements and higher converged. FE simulations were then used in ABAQUS/EXPLICIT [42] to further analyze the different combinations of water and dry brain, similar to the way a specimen would undergo deformation during a SHPB experiment. The specimen geometry used for conducting FE simulations was cylindrical with a radius of 15 mm. The thickness of the cylindrical specimen was 15 mm.

## 3. Results and Discussion

### 3.1. Experiment Response

**Figure 7:**
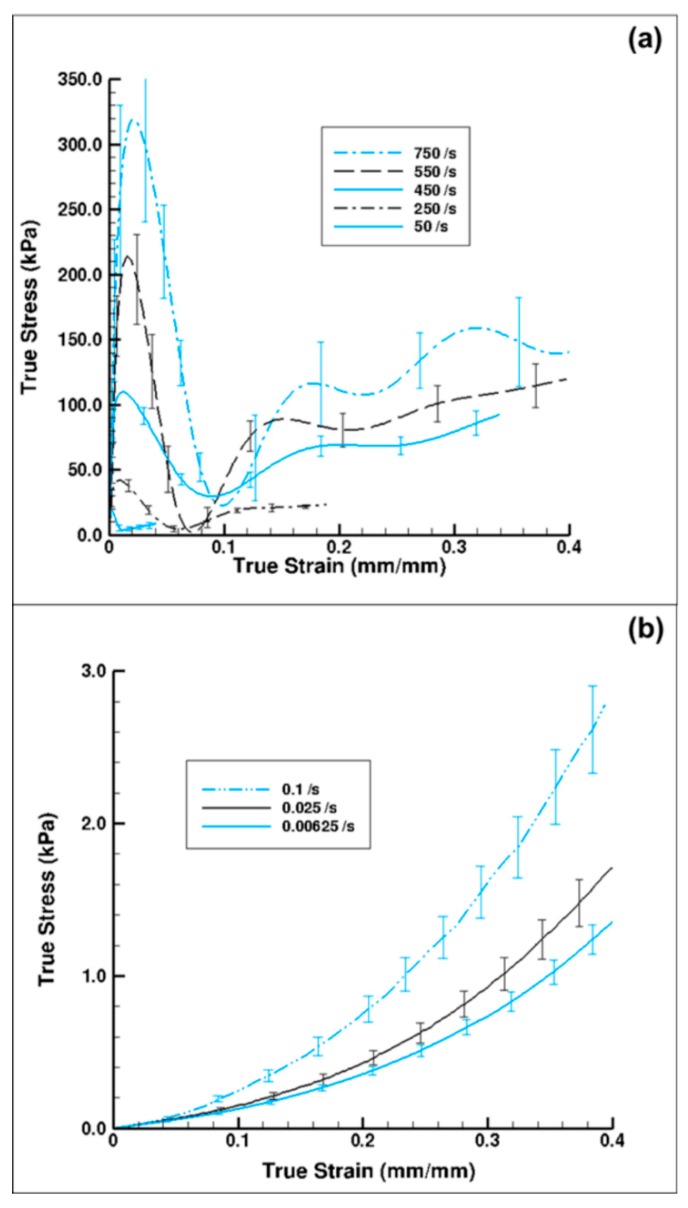
Comparison of (**a**) high strain rate (n = 4 (750 s^−1^), 7 (750 s^−1^), 5 (750 s^−1^), 4 (750 s^−1^), and 6 (750 s^−1^)) [29] and (**b**) quasi-static strain rate wet-brain material behavior (n = 16 (0.1 s^−1^), 7 (0.025 s^−1^), and 6 (0.00625 s^−1^)). The water content in the wet brain sample was ~80% m/m. Note the lower stress values for the quasi-static wet brain case and that the initial peak in stress observed under high-rate loading was not present in the quasi-static loading. The error bars represent standard error.

Figure 7 shows plots of the experimental true stress–strain behavior of the wet porcine brain at both high and quasi-static strain rates under compression, which exhibited two distinct patterns of stress–strain behaviors. We also note that both the low- and high-rate testing methods showed significant strain-rate sensitivity.

**Figure 8:**
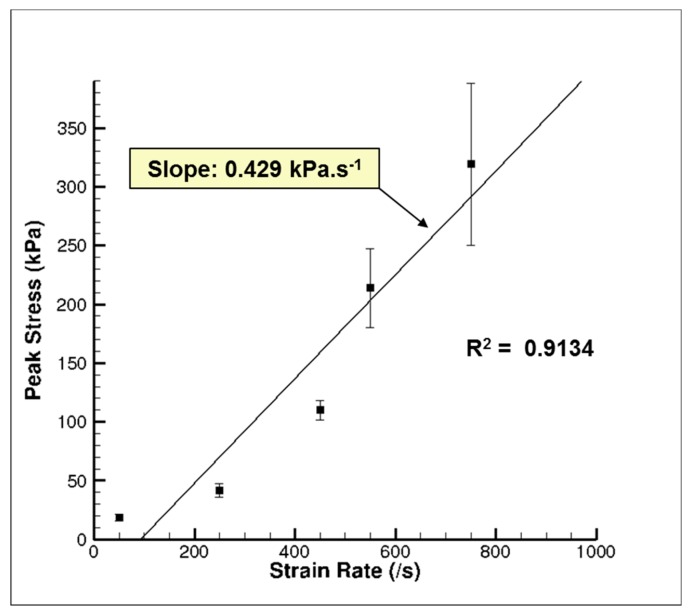
Plot of the initial hardening peak stress, σ_p_, of the wet porcine brain from the high strain rate tests ranging from 50–750 s^−1^ [29]. The water content in the porcine brain was ~80% m/m. Twenty-six brain samples were analyzed. The error bars represent standard error.

The high-rate experimental data showed an initial hardening trend similar to the yield point in some high carbon steel alloys or thermoplastics [40], followed by softening and then further hardening at higher strains (Figure 7a). A similar high-rate phenomenon was also reported by Sparks and Dupaix [26], Prabhu [29], and Clemmer [30]. These initial hardening and softening trends were highly strain-rate dependent, thus illustrated by the initial hardening peak stress, σ_p_. σ_p_ also marked the transition from elastic to inelastic deformations. We also observed that the strains corresponding to σ_p_ increased steadily as the strain rate increased (Figure 7a). Hence, σ_p_ occurred at different true strain values for strain rates of 50, 250, 450, 550, and 750 s^−1^. Figure 8. represents a plot of σ_p_ versus different strain rates in the high-rate regime and shows that σ_p_ increased linearly as the strain rate increased. A one-way analysis of variance (ANOVA) method for statistical analyses on the tangent modulus, σ_p_ and the true strain at σ_p_ was conducted by Prabhu [29]. They showed that there was a significant difference for σ_p_ and the true strain at σ_p_ over the strain rates 50–750 s^−1^, with *p* values <0.05. However, no statistical difference was observed for the tangent modulus. 

**Figure 9:**
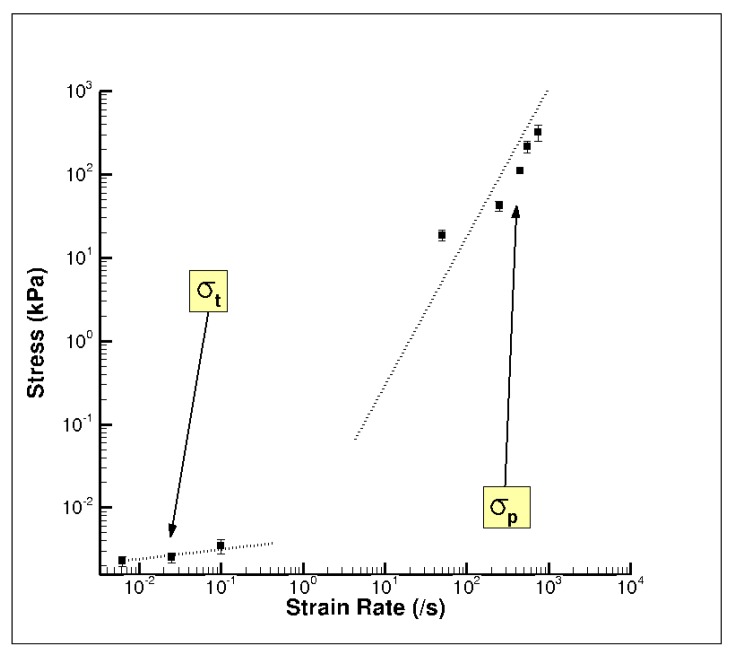
Comparison of the elastic–inelastic transition stress, σ_t_, and the initial hardening peak stress, σ_p_, of the wet porcine brain at varying strain rates using a log–log scale. The water content of the wet porcine brain was ~80% m/m. All 39 brain samples were analyzed. The error bars represent standard error.

In contrast, the quasi-static wet brain tissue behavior followed a monotonic increase indicative of material hardening, which is a stress–strain relation more typically exhibited by soft tissues (Figure 7b), such as seen in muscle, brain, liver, and tendon tissues [23,38,43]. Unlike the high-rate behavior, at quasi-static rates, the material (wet brain) continues to harden after the initial elastic response with the mechanical behavior at the quasi-static regime being completely devoid of the initial hardening and softening trend noted in high-rate response. However, the elastic–inelastic transitions for both quasi-static and dynamic data occur at similar strain levels (Figure 7). At quasi-static rates, if one were to consider the elastic-viscoelastic transition stress, σ_t_, at the true strain values signifying elastic-viscoelastic transition (analogous to the high strain rate σ_p_ marking elastic–inelastic transition), the variation of σ_t_ over quasi-static strain rates was observed to be nominal (Figure 9).

Table 5 presents the variation of the mean σ_t_, the strain at σ_t_, and the tangent modulus at 0.01 true strain over quasi-static strain rates; along with *p* values. With *p* < 0.05, a statistical difference in tangent modulus was noted over the quasi-static strain rates, but no statistical difference was observed for σ_t_ and the true strain at σ_t_. In other words, Figure 9 and Table 5 illustrate the rather soft variance of quasi-static σ_t_ values. While σ_t_ marks the elastic–inelastic transition leading to subsequent hardening of the material, σ_p_ marks the linear-nonlinear transition followed by a softening trend. A comparison of σ_t_ at quasi-static rates and σ_p_ at high strain rates indicates the presence of two different deformation mechanisms that are highly dependent on temporal rates (Figure 7 and Figure 9; Table 5). The deformation mechanism at quasi-static rates was initially marked by an elastic regime that can be explained by the percolation of water through the specimen matrix [25,43]. The lower rates of the quasi-static regime are favorable to the slow migration of water through the various intercellular cavities in the matrix. At higher strains, the percolation of water is constrained due to the reducing specimen volume and the consequential tissue (cellular structure and matrix) compaction. The hardening trend observed at higher strains could be attributed to the resistance offered by the tissue as it compacts.

In comparison, the wet brain samples exhibited an initial mechanical response when tested at dynamic rates that are not observed in quasi-static tests (Figure 7). Song [27] suggested this initial mechanical response is solely due to radial inertial effects in soft materials, but Prabhu [29] asserted that at least a portion of this initial response is an intrinsic material property. If the observed initial mechanical response is due to the tissue being deformed faster than water can easily percolate out of the cells, some or all of the behavior would indeed be intrinsic to the material. In other words, the water present in the matrix and cellular structures offers inertial resistance to the sudden deformation, giving rise to a sharp initial mechanical response. The initial hardening trend is then followed by a softening trend is due to the rupturing of cells. Further compaction leads to the realignment of damage cellular and matrix components, producing a strain hardening effect at higher strains. 

**Figure 10:**
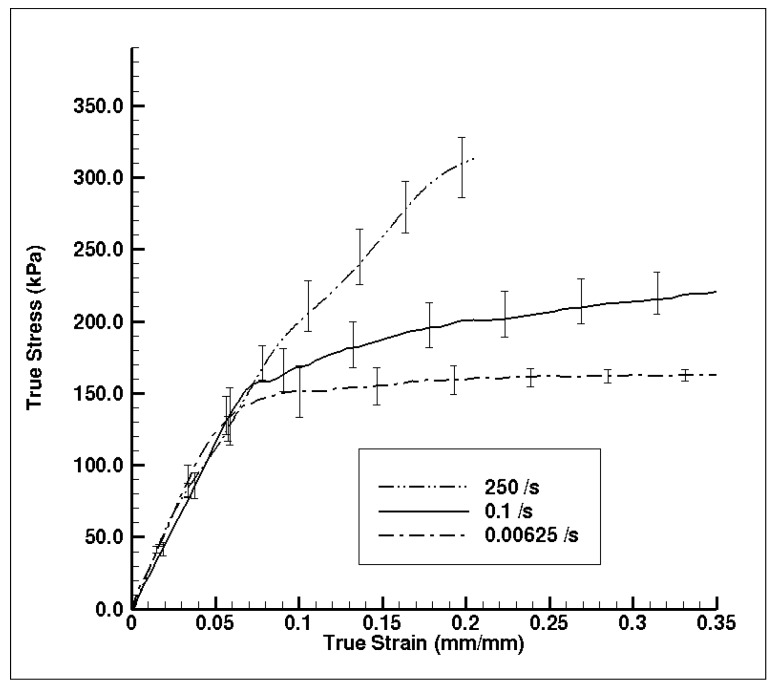
Comparison of experimental quasi-static and high strain rate dry brain (~0% water content m/m) stress–strain behavior (n = 4 (250 s^−1^), 5 (0.1 s^−1^), and 4 (0.00625 s^−1^)). Specimens possessed a diameter of 15 mm diameter and a thickness of 5 mm. The error bars represent standard error.

Figure 10 shows the experimental true stress–strain mechanical response of dry porcine brain under compression at the quasi-static strain rates of 0.00625 and 0.1 s^−1^ and the high strain rate of 250 s^−1^. The wet brain behavior in Figure 7b was akin to most soft biological materials at quasi-static rates, which exhibit a toe region at the beginning, then an intermediate inelastic behavior, and finally a hardening at larger strains. However, the dry brain behavior in Figure 10 was more similar to the quasi-static behavior of metals, with an initial elastic region followed by a work hardening regime. Such contrasting trends in the material behaviors can be attributed to the presence of water in the wet brain (~80%) [25,29], which contributes to the strain rate sensitivity for both quasi-static and high rates. For the dry brain, Table 6 shows that an absence of water makes the tangent modulus, yield point (elastic–inelastic transition stress), and strain at yield point in the stress–strain behavior insensitive to the applied strain rate.

The characteristic of the dry brain true stress–strain behavior is similar at the three applied strain rates that were employed (Figure 10); the stress–strain behavior was marked by an initial toe region with an elastic response up to a ‘yield point’ and then followed by a work hardening at larger strains. As noted from Figure 10, the variation in yield point did not change as the applied strain rate changed, although the work hardening increased (concave-down) as the applied strain rate increased. Table 6 presents the results of the statistical analyses on the dry brain tangent modulus, elastic–inelastic transition stress (σ_p_/σ_t_) or stress at yield point, and the true strain at σ_p_/σ_t_. The *p*-value for all three parameters was greater than 0.05 at quasi-static and high strain rates, implying statistical indifference. Hence, the tangent modulus, yield point (elastic–inelastic transition stress), and strain at yield point of the true stress–strain behavior for the dry brain was observed to be strain rate insensitive. The similarities between the quasi-static and dynamic rates for the dry brain in Figure 10 highlight the effect of water on the mechanical response of brain tissue. Since there is minimal difference between the quasi-static and dynamic mechanical results for dry brain prior to the yield point, much of the observed initial mechanical response is due to water contained in the sample. This finding reinforces the above supposition that the initial mechanical response observed for the wet brain when tested at dynamic rates is due to water being forced out of the tissue matrices and cellular structures faster than the water can easily percolate. Therefore, future testing must consider how much the initial mechanical response is due to radial inertial effects and how much is a true tissue mechanical response.

### 3.2. Simulation Response

**Figure 11:**
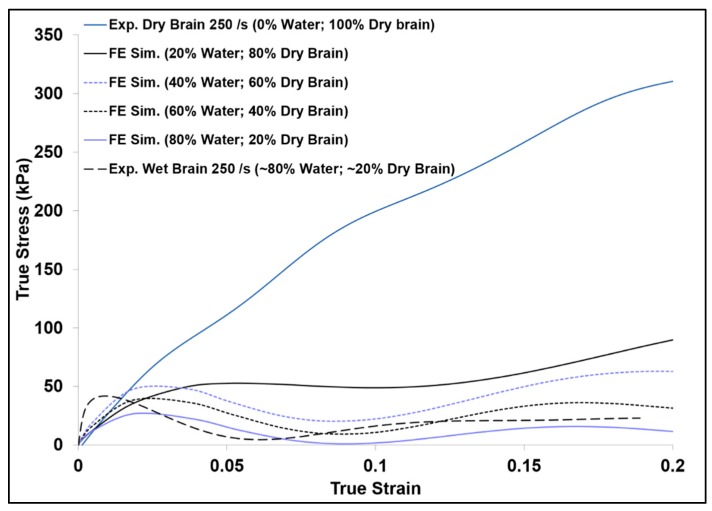
Comparison of finite element (FE) simulation and experimental true stress–strain (σ_33_) behavior of specimens with various water and dry brain m/m contents at a strain rate of 250 s^−1^. Here compressive stress and strain are taken as positive.

As further investigations were performed on the tissue hydration effects at high strain rates using finite element analysis (FEA), the FE simulation results showed a substantial increase of the initial hardening trend as the strain rate increased for wet brain material (Figure 7a). Figure 11 shows the FE simulation of the various proportions of water (20%, 40%, 60%, and 80% m/m) and the dry brain (80%, 20%, 40%, and 60% m/m) along with the experimental high strain rate response of the wet (~80% water) and the dry brain (~0% water) at 250 s^−1^. Examining the FE simulation results in Figure 11 shows that the trend of the FE simulation true stress–strain curves change as the m/m content of water and the dry brain were changed. The specimen with the lowest water content (20% m/m) had the highest stress, and the observed stress became lower as the m/m water content in the specimen was increased. In other words, the initial stress response in the high-rate response of the specimen was inversely proportional to the tissue hydration at high strain rates. This behavior is unlike the brain tissue response reported by Cheng and Bilston [31], where the creep rate of the brain white matter at quasi-static rates depended on the water movement out of the sample. At high strain rates, the water resistance to rapid movement is distinctly seen in the initial response of the FE specimens with larger m/m water content. Furthermore, as the m/m water content in the sample increased the initial hardening trend also increased. This again validates the assertion that cellular cavity water content plays a significant role in the inertial effects of the brain tissue. The greater the hydration of the brain tissue the higher the initial hardening trend is within the high strain rate mechanical response. Additionally, the experimental results shown in Figure 11 give the upper and lower bounds for the FE simulation results as they contain the lowest and highest amount of water. 

Results for the current experimental and computational investigation show that water plays an important role in the mechanical response of brain tissue at all strain rates. Furthermore, it can be inferred that water plays a crucial role in the strain rate sensitivity and deformation of the brain. Prior studies by Prabhu [29] and Clemmer [30] have shown that the presence of water in matrix and cells notably influences the stress-state at quasi-static and high strain rates. Weinberg and Ortiz [44] showed that the cavitation of water at high strain rates causes biological damage to surrounding tissue. This behavior needs to be included in material models and accounted for when simulating human brain injury scenarios. 

This behavior can be implemented into brain tissue material models to better design protective gear for use in dynamic rate conditions by incorporating the effects of water into brain constitutive material models. Including this data has the potential to better capture the brain’s mechanical response to trauma [45] and to generate higher fidelity finite element simulation responses. These simulations could benefit the design of protective safety equipment, such as helmet designs [46], because it would allow quickly iterating through designs and materials to optimize protective headgear and include how the brain would be affected. 

Furthermore, these findings have implications in the way that brain tissue, and perhaps some other soft materials, need to be examined at dynamic rates with regards to the initial mechanical response. Distinguishing between inertial effects and inherent water-related material properties will provide a better understanding of the brain’s micro and macro mechanics, which can then be potentially linked to physiological damage. This difference affects the way that brain tissue is considered and analyzed at dynamic rates as well as the way material models for brain should be implemented in macroscale simulation models. The current study provides a novel description for the effect of hydration level on the mechanical behavior of the brain. It also lays the experimental basis for a mixture theory-based material modeling of the brain. In extension, this change in the simulated material model will affect the way macroscale models of the brain will respond to dynamic deformations and any protective headgear design they are used for. Hence, quantifying the role of water at these strain rate ranges could help unlock a better understanding of the physics and pathophysiology of TBI. Lastly, this study is limited without histological quantification of water’s role during deformation, a process that will constitute the scope of future research.

## 4. Conclusions

The goal of this research was to assess hydration effects on the mechanical behavior of porcine brain over a range of strain rates (quasi-static and high dynamic rates) and analyze the tissue hydration effects using FEA at high strain rates. Experimental results show a strong strain rate dependence for the wet brain (~80% m/m); however, the dry brain’s tangent modulus, yield point (elastic–inelastic transition stress), and strain at yield point were strain rate insensitive (Figure 7 and Figure 10). Two different phenomenological behaviors emerged at the different applied strain rates as well. At higher strain rates (~700 s^−1^), the wet brain’s behavior was marked by an initial hardening trend, followed by a strain-softening, and then strain-hardening (concave-up) as the deformation increased. In contrast at quasi-static strain rates (~0.01 s^−1^), the wet brain’s behavior was marked by an initial toe region and then by concave-up strain hardening similar to other soft tissue phenomenological behavior [47]. The tangent modulus, initial hardening peak stress (σ_p_) and strain at σ_p_ of the wet brain high strain rate data showed a statistical significance (Figure 8 and Table 5) with inconsequential rate dependence of elastic–viscoelastic transition stress (σ_t_) and strain at σ_t_ in the quasi-static range (Figure 9 and Table 5).

At quasi-static rates, the difference between wet (Figure 7b) and dry (Figure 10) brain material was significant in terms of the magnitude of the stresses and the characteristic stress–strain behavior. The dry brain material incurred concave-down hardening while the wet brain incurred concave-up hardening. Significant differences in σ_t_ and strain at σ_t_ over quasi-static strain rates point to distinct characteristics of the mechanical responses of wet and dry brain (Table 5 and Table 6); however, no significant difference was observed in the tangent modulus, σ_t_, and strain at σ_t_ of the dry brain at quasi-static and higher strain rates (Table 6). Micromechanical FEA using various proportions of water in the dry brain further showed that water played a major role on the initial hardening trend (Figure 11). In summary, the different mechanical properties and behavior trends could be attributed to water’s dominant role at quasi-static and high rates. As such, the need to develop constitutive models with the effect of water is crucial to the understanding of the physics and pathophysiology of TBI.

## Figures and Tables

**Table 1:** Details of samples, animal brain numbers, and testing conditions at each strain rate

Strain Rate (s^−1^)	Wet/Dry	Number of Samples	Number of Animals/Porcine Brains	Temperature (°C)	Pressure (MPa)
**0.00625**	Wet	6	3	20.85	0.1
**0.025**	Wet	7	4	20.85	0.1
**0.1**	Wet	16	7	20.85	0.1
**50**	Wet	6	3	20.85	0.1
**250**	Wet	4	2	20.85	0.1
**450**	Wet	5	2	20.85	0.1
**550**	Wet	7	3	20.85	0.1
**750**	Wet	4	2	20.85	0.1
**0.00625**	Dry	4	2	20.85	0.1
**0.1**	Dry	5	3	20.85	0.1
**250**	Dry	4	2	20.85	0.1
**Total**		68	33		

**Table 2:** Overview of the finite element (FE) simulation cases for the micromechanics of dry brain and water content variation.

FE Simulation Case	Dry Brain % (m/m)	Water % (m/m)
1	80	20
2	60	40
3	40	60
4	20	80

**Table 3:** Summary of the model equations for MSU TP 1.1 (see Bouvard et al. [40]). See Appendix C for a summary of symbol descriptions.

Term/Function	Description
$\bar{\Psi }=\bar{\Psi }\left({\bar{C}}^{e},{\bar{\xi }}_{\;1},{\bar{\xi }}_{2},\right)$, where ${\bar{C}}^{e}$ is the elastic part of the right Cauchy-Green tensor, and ${\bar{\xi }}_{1}$, ${\bar{\xi }}_{2}$ and ${\bar{E}}^{\bar{\beta }}$ are internal strain fields (internal state variables).	Free energy, $\bar{\Psi }$
$\sigma =J^{e-1}\tau =J^{e-1}F^{e}\bar{S}F^{eT}$, where J^e-1^ is the inverse of the determinant of $F^{e}$. $F^{e}$ and $F^{eT}$ are the elastic part of F and the transpose of elastic part of **F**. $\bar{S}=2\frac{\partial \hat{\bar{\Psi }}}{\partial {\bar{C}}^{e}}$	Cauchy Stress, σ Second Piola-Kirchhoff Stress, $\bar{S}$
$\tau_{1}=R^{e}{\bar{M}}_{1}R^{eT}$, where $R^{e}$ and $R^{eT}$ are the elastic part of the rotation tensor (R) and the transpose of the elastic part of R. $\bar{M}=2\mu {\bar{E}}^{e}+\left(K-\frac{2}{3}\mu \right)\bar{\mathrm{Tr}}\left({\bar{E}}^{e}\right)\bar{I}$, where μ and K are the elastic shear and bulk moduli modeling the elastic behavior respectively. $E^{e}$ is the elastic part of the Green-Lagrange strain tensor, and $\bar{I}$ is the identity matrix. $F=F^{e}F^{p},F^{e}=R^{e}U^{e},{\bar{E}}^{e}=\ln(U^{e})$, where $F^{p}$ is the plastic part of the deformation tensor, and $U^{e}$ is the right stretch tensor.	Kirchhoff Stress (elasto-viscoplastic part, $\tau_{l}$) Elastic Law (Mandel Stress, $\bar{M}$) Deformation Gradient
${\bar{\kappa }}_{1}=\frac{\partial \hat{\bar{\Psi }}}{\partial {\bar{\xi }}_{\;1}}$, ${\bar{\kappa }}_{2}=\frac{\partial \hat{\bar{\Psi }}}{\partial {\bar{\xi }}_{2}}$, where ${\bar{\kappa }}_{1}$ and ${\bar{\kappa }}_{2}$ are stress-like thermodynamic conjugates of the ${\bar{\xi }}_{1}$ and ${\bar{\xi }}_{2}$ respectively. $\bar{\alpha }=\frac{\partial \hat{\bar{\Psi }}}{\partial {\bar{E}}^{\bar{\beta }}}$, where $\bar{\alpha }$ is a stress-like thermodynamic conjugate of ${\bar{E}}^{\bar{\beta }}$.	Stress-like internal state variables Stress-like internal state variable
${\dot{F}}^{p}={\bar{D}}^{p}F^{p}$, where ${\bar{D}}^{p}$ is the inelastic rate of deformation. ${\bar{D}}^{p}=\frac{1}{\sqrt{2}}{\dot{\bar{\gamma }}}^{p}\;{\bar{N}}^{p}$ with ${\bar{N}}^{p}=\frac{\bar{\mathrm{DEV}}\left({\bar{M}}_{1}\right)}{\left\|\bar{\mathrm{DEV}}\;({\bar{M}}_{1})\right\|}$, where ${\dot{\bar{\gamma }}}^{p}$ is the viscous shear strain rate given by the following equation: ${\dot{\bar{\gamma }}}^{p}={\dot{\bar{\gamma }}}_{0}^{p}{\left[\sinh\left(\frac{\langle \bar{\tau }-\left({\bar{\kappa }}_{1}+{\bar{\kappa }}_{2}+\alpha_{p}\bar{\pi }\right)\rangle }{Y}\right)\right]}^{m}$ with $\bar{\tau }=\frac{1}{\sqrt{2}}\|\bar{\mathrm{DEV}}\left(\bar{M}-\bar{\alpha }\right)\|$ and $\bar{\pi }=-\frac{1}{3}\bar{\mathrm{Tr}}\left(\bar{M}\right)$, where $\bar{\tau }=\left(1/\sqrt{2}\right)\|\bar{\mathrm{DEV}}\left(\bar{M}-\bar{\alpha })\right\|$ is an equivalent shear stress term and $\bar{\pi }=\left(1/3\right)\bar{\mathrm{Tr}}\left(\bar{M}\right)$ is the effective pressure term, ${\dot{\bar{\gamma }}}_{0}^{p}$ is a reference strain rate, m is a strain rate sensitivity parameter, and αp is a pressure sensitivity parameter and Y is the yield criterion. ${\dot{\bar{\xi }}}_{\;1}=h_{0}\left(1-\frac{{\bar{\xi }}_{\;1}}{{\bar{\xi }}^{*}}\right){\dot{\bar{\gamma }}}^{p}$, ${\dot{\bar{\xi }}}^{\;*}=g_{0}\left(1-\frac{{\bar{\xi }}^{\;*}}{{\bar{\xi }}_{\mathrm{sat}}^{*}}\right){\dot{\bar{\gamma }}}^{p}$, where $\xi^{*}$ represents an evolving strain threshold or criterion that the macromolecular chains must overcome to slip. h_0_ and g_0_ are hardening moduli, and $\xi_{\mathrm{sat}}^{*}$ is the saturation value of $\xi^{*}$. ${\dot{\bar{\xi }}}_{\;2}=h_{1}\left({\bar{\lambda }}^{p}-1\right)\left(1-\frac{{\bar{\xi }}_{\;2}}{{\bar{\xi }}_{\;2\mathrm{sat}}}\right){\dot{\bar{\gamma }}}^{p}$ with ${\bar{\lambda }}^{p}=\frac{1}{\sqrt{3}}\sqrt{\bar{\mathrm{Tr}}\left({\bar{B}}^{p}\right)}$ and ${\bar{B}}^{p}=F^{p}F^{pT}$ where $h_{1}$ is the hardening modulus, and $\xi_{2\mathrm{sat}}$ is the saturation value of ξ_2_. $\dot{\bar{\beta }}=R_{s_{1}}\left({\bar{D}}^{p}\bar{\beta }+\bar{\beta }\;{\bar{D}}^{p}\right)$ and $\bar{\beta }\left(X,0\right)=I$	Flow rule Equivalent plastic shear strain-rate Polymer chain resistance to plastic flow Polymer chain crystallization at large strain Evolution equation of stretch-like tensor $\bar{\beta }$
$\left\{\mu ,\;K,{\dot{\gamma }}_{0}^{p},\;m,\;Y\right\}$ $\left\{{\bar{\xi }}_{0}^{*},{\bar{\xi }}_{\mathrm{sat}}^{*},{\;h}_{0},{\;g}_{0},C_{\kappa_{1}}\right\}$ $\left\{h_{1},{\bar{\xi }}_{\;2\mathrm{sat}},{\;C}_{\kappa_{2}}\right\}$ $\left\{R_{S1},\lambda_{L},\mu_{R}\right\}$ $\left\{\alpha_{p},{\bar{\xi }}_{10},{\bar{\xi }}_{20}\right\}$	Material constants

**Table 4:** Values of material constants for dry brain material using MSU TP Ver. 1.1 viscoplasticity model along with their definitions.

Model Constants	Constant Definition	Values
μ (MPa)	Shear Modulus	0.80
K (MPa)	Bulk Modulus	399.73
${\dot{\bar{\gamma }}}^{p}$ (s^−1^)	Reference Strain Rate	120,000
m	Strain Rate Sensitivity Parameter	0.90
Y_o_ (MPa)	Material Yield Parameter	9.00
α_p_	Sensitivity Parameter	0
λ_L_	Network Locking Stretch	2.00
μ_R_	Rubbery Modulus	0.07
R_s1_	Material Hardening Parameter	1.4
h_o_	Hardening Modulus	0.41
ξ^o^_1_	Internal Strain-Like Parameter Initial Value	0.0045
ξ^*^_sat_	Internal Strain-Like Parameter Saturation Value	0.001
ξ^*^_o_	Energetic Strain Barrier	1.2
g_o_	Hardening Modulus	0.3
Cκ_1_ (MPa)	Internal Stress-Like Parameter	0.41
h_1_	Hardening Modulus	0
e^o^_s2_	Internal Strain-Like Parameter Initial Value	0
e^sat^_s2_	Internal Strain-Like Parameter Saturation Value	0.4
Cκ_2_ (MPa)	Internal Stress-Like Parameter	0

**Table 5:** Statistical comparison of the tangent modulus, elastic–inelastic transition stress (σ_t_) and strain at σ_p_/σ_t_ of the wet brain (~80% m/m water content) at quasi-static and high strain rates. *P* < 0.05: significantly different; *p* > 0.05: significantly indifferent.

	Strain Rate (s^−1^)	0.00625 s^−1^ n = 6	0.0250 s^−1^ n = 7	0.100 s^−1^ n = 16	*p*-Value
Variable					
Tangent Modulus (kPa)		1.6822 ± 0.0047	1.7497 ± 0.0021	3.2378 ± 0.0371	*<0.05*
Transition Stress (kPa)		0.1046 ± 0.0046	0.1069 ± 0.0020	0.1400 ± 0.0377	*>0.05*
Strain at Peak Stress		0.075 ± 0.0040	0.075 ± 0.0060	0.072 ± 0.0050	*>0.05*

**Table 6:** Statistical Comparison of the tangent modulus, elastic–inelastic transition stress (σ_p_/σ_t_) and strain at σ_p_/σ_t_ of the dry brain (~0% m/m water content) at quasi-static and high strain rates. *p* < 0.05: significantly different; *p* > 0.05: significantly indifferent.

	Strain Rate (s^−1^)	0.00625 s^−1^ n = 4	0.100 s^−1^ n = 5	250 s^−1^ n = 4	*p*-Value
Variable					
Tangent Modulus (kPa)		2591.451 ± 424.4080	2282.160 ± 922.3810	2143.683 ± 620.0000	>0.05
Transition Stress (kPa)		108.602 ± 17.7860	138.313 ± 55.9020	166.389 ± 48.1420	>0.05
Strain at Transition Stress		0.0419 ± 0.0086	0.0606 ± 0.0100	0.0776 ± 0.0106	>0.05

**Figure A1:**
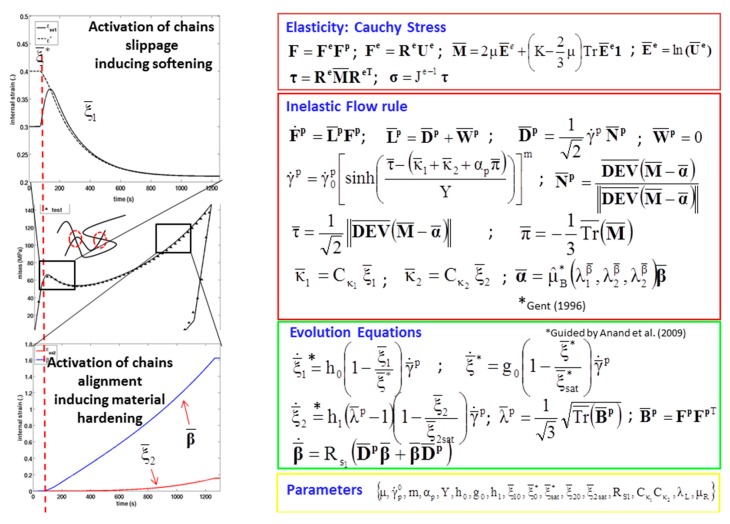
Overview of the material model, MSU TP Ver. 1.1, with salient features of the flow rule and evolution equations [40].

**Figure A3:**
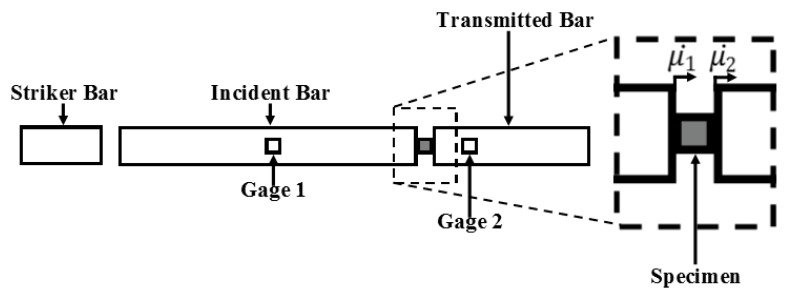
Illustration showing the location for the bars, strain gauges, and specimen in a typical Split-Hopkinson pressure bar (SHPB) configuration. Velocities for the incident and transmitted bars are denoted by $\dot{u_{1}}\mathrm{and}$ $\dot{u_{2}}$.

## Appendix A

The material model, MSU TP Ver. 1.1, has been developed to account for the elastic and inelastic deformation of an amorphous polymer. The kinematics of the material model draws its inspiration from the works of Kröner [48] and Lee [49] in which a multiplicative decomposition of the deformation gradient **F** (into elastic and inelastic components) was proposed. This multiplicative decomposition renders itself to an elastoviscoplastic model. The expression for **F** is given as

(A1) $$F=F^{e}F^{p}\;,\;J=\det F={\;J}^{e}J^{p}\;,{\;J}^{e}=\det\;F^{e}\;,{\;J}^{p}=\det\;F^{p}$$

where J is the determinant of **F**. Such a description is physically motivated by the mechanisms underlying the elasticity and inelasticity in amorphous polymers. $F^{e}$ represents the elastic part due to ‘reversible elastic mechanisms’, such as bond stretching and mainly chain rotation inducing the different conformations of the intermolecular structure in the polymeric material. $F^{p}$ represents the inelastic or plastic part due to ‘irreversible mechanisms’, such as permanent chain stretching and the dissipative mechanisms due to the relative slippage of molecular chains in polymers. An overview of the material model with representative elastic and inelastic parts is given in Figure A1.

The kinematic multiplicative decomposition (Equation (A1)) suggests that there exists an intermediate configuration between the undeformed **B_o_** and the current **B** configuration, which is denoted here by $\bar{B}\;$. All calculations of the inelastic flow and evolution equations were performed in this intermediate configuration $\bar{B}$. The Clausius–Duhem inequality [50] is then prescribed in Equation (A2).

(A2) $$\tau :d_{e}+\;\bar{M}:\;{\bar{D}}_{p}-\dot{\bar{\psi }}\geq 0$$

Where **τ** is the Cauchy stress in the current configuration (**B**), ${\bar{D}}^{e}$ is the elastic rate of deformation tensor, $\bar{M}$ is the Mandel stress residing in the intermediate configuration ($\bar{B}\;$), ${\bar{D}}^{p}$ is the plastic rate of deformation tensor, and $\dot{\bar{\psi }}$ is the rate of change of the strain energy density function. Inelastic flow was of the model was assumed to be incompressible, and was void of plastic rotational component, that is, ${\bar{w}}^{p}=0$ [50,51]. The flow rule for the inelastic deformation captures the polymer’s viscous flow observed during the yielding of the material. The inelastic deformations normally arise from the localized slips and viscous flow processes of polymeric chains that result in permanent displacement. In order to capture the rate of change of such processes, the flow rule represented by Equation (A3).

(A3) $${\dot{F}}^{p}={\bar{D}}^{p}F^{p},\;{\bar{D}}^{p}=\left(1/\sqrt{2}\right).{\dot{\bar{\gamma }}}^{p}{\bar{N}}^{p}$$

where ${\bar{N}}^{p}=\frac{\bar{DEV}\;\left(\bar{M}-\bar{\alpha }\right)}{\|\bar{DEV}\left(\bar{M}-\bar{\alpha })\right\|}$ is the direction of the viscous flow, ${\bar{D}}^{p}$ the inelastic rate of deformation, and ${\dot{\bar{\gamma }}}^{p}$ is the viscous shear strain rate given by the equation

(A4) $${\dot{\bar{\gamma }}}^{p}={\dot{\bar{\gamma }}}_{0}^{p}{\left[Sinh\;\left(\frac{\bar{\tau }-\left({\bar{\kappa }}_{1}+{\bar{\kappa }}_{2}+\alpha_{p}\bar{\pi }\right)}{Y}\right)\right]}^{m}$$

where $\bar{\tau }=\left(1/\sqrt{2}\right)\|\bar{DEV}\left(\bar{M}-\bar{\alpha })\right\|$ is an equivalent shear stress term and $\bar{\pi }=\left(1/3\right)\bar{Tr}\left(\bar{M}\right)$ is the effective pressure term. In Equation (A4), m is the strain rate sensitivity parameter, Y is a material parameter, and $\alpha_{p}$ is the pressure sensitivity parameter. Equation (A4) has been derived from previous works on plastic flow rules by Fotheringham and Cherry [52], Anand [53], and Richeton [54,55]. The inverse hyperbolic sine function captures inelastic material response that leads to thermal dissipation, and m corresponds to the nature of the motion of polymeric chains.

${\bar{\xi }}_{1}$, ${\bar{\xi }}_{2}$, and ${\bar{E}}^{\bar{\beta }}$ are internal strain fields (internal state variables) induced due by the presence of defects such as bio-polymeric chain locking or entanglement of macromolecular chains. They are associated with the irreversible mechanisms related to the inelastic material behavior. While ${\bar{\xi }}_{1}$ captures the strain-like quantity arising due to polymeric chain locking and entanglement, ${\bar{\xi }}_{2}$ represents the internal strain-like quantity induced from the alignment of polymeric chains. Additionally, ${\bar{E}}^{\bar{\beta }}$ is a tensorial variable associated with the stretch-like tensor $\bar{\beta }$, which represents directional-dependent hardening induced at large deformations by the stretching of polymeric chains and fibers. Furthermore, ${\bar{\kappa }}_{1}$, ${\bar{\kappa }}_{2}$, and $\bar{\alpha }$ are stress-like thermodynamic conjugates of the ${\bar{\xi }}_{1}$, ${\bar{\xi }}_{2}$, and ${\bar{E}}^{\bar{\beta }}$ respectively. Additionally, ${\bar{\xi }}^{*}$ corresponds to the strain threshold (corresponding to an energetic barrier) after which the polymeric chains can slip. In the evolution equations of the internal state variables pertaining to inelastic deformation, h_o,_ h_1,_ and g_o_ are the hardening moduli for the internal strain-likes terms ${\bar{\xi }}_{1}$, ${\bar{\xi }}_{2}$, and ${\bar{\xi }}^{*}$ respectively. Here ${\bar{\xi }}_{2\mathrm{sat}}$ and ${\bar{\xi }}_{\mathrm{sat}}^{*}$ relate to the saturation values of ${\bar{\xi }}_{2}$ and ${\bar{\xi }}^{*}$. As mentioned above, ${\bar{E}}^{\bar{\beta }}$, which arises from the stretching of polymeric chains and fibers at large deformation was derived using statistical approach [56] for hyperelasticity. Here ${\hat{\mu }}_{B}$ represents the modulus of internal shear stress tensor $\bar{\alpha }$. The evolution of $\bar{\beta }$ follows a kinematic hardening-type rule for metals that was first proposed by Prantil et al. [57] and then adopted by Ames et al. [58] for polymers, and $R_{s_{1}}$ is a constant specific to the material.

A one-dimensional version (material point simulator) of the above-mentioned material model was implemented in the MATLAB [41], and then the experimental data was used to calibrate the material point simulator using optimization functions available in MATLAB (Figure A2). Once the material point simulator and the experimental data had correlation, the derived material parameters were then implemented in ABAQUS/Explicit [42] through a three-dimensional user material subroutine to describe the material’s mechanical response.

## Appendix B

Split-Hopkinson pressure bar (SHPB) is a high strain rate testing apparatus comprised of a series of bars, namely: the striker bar, incident bar, and transmitted bar. These bars transmit a single shock wave, provided by the impact of the striker bar, through a specimen sandwiched between the incident and transmitted bars. This shock wave is useful for gathering stress strain relations during high strain rate deformation. An illustration of the SHPB apparatus is shown in Figure A3.

The theory of an SHPB is based on the elastic wave propagation in long cylindrical bars, and on the principle of superposition of waves. The stress wave traveling through the bar is assumed to propagate longitudinally with viscoelastic dispersion, due to our use of polycarbonate as the bar material. The initial wave, the incident wave, propagates down the incident bar and is recorded by a strain gauge attached to the incident bar, known as gauge 1. Due to the specimen having different mechanical impedance than the bars, a portion of the incident wave is transmitted through the specimen, while the remainder of the wave is reflected off of the specimen-bar interface. The transmitted portion, known as the transmitted wave, is captured by a strain gauge located on the transmitted bar. The reflected wave is captured by gauge 1.

The velocities of the bars near the specimen-bar interfaces,$\dot{\;u\;}$, at some time, *t*, are shown to be

(A5) $$\dot{u_{1}}\left(t\right)=-c_{1}*\left(\varepsilon_{i}\left(t\right)-\varepsilon_{r}\left(t\right)\right)$$

(A6) $$\dot{u_{2}}\left(t\right)=-c_{2}*\varepsilon_{t}\left(t\right)$$

where $c_{1}$ is the longitudinal wave speed, *ɛ* is the strain gauge record, and the subscripts *i*, *r*, and *t* are the incident, reflected, and transmitted waves, respectively. Subscripts 1 and 2 represent the incident and transmitted bars and are illustrated in Figure A3. By knowing the velocities of the bars at the specimen–bar interfaces, the strain rate,$\;\dot{\varepsilon_{s}}$, and strain $\varepsilon_{s}$, of the specimen can be found as

(A7) $$\dot{\varepsilon_{s}}\left(t\right)=\frac{\dot{u_{1}}\left(t\right)-\dot{u_{2}}\left(t\right)}{L_{s}}$$

(A8) $$\varepsilon_{s}\left(t\right)=\int^{\text{​}}\dot{\varepsilon_{s}}\left(t\right)dt$$

where *L* is the instantaneous length and the subscript s refers to the specimen. The forces, *F*, in the bars can then be found:

(A9) $$F_{1}\left(t\right)=A_{1}E_{1}*\left(\varepsilon_{i}\left(t\right)+\varepsilon_{r}\left(t\right)\right)$$

(A10) $$F_{2}\left(t\right)=A_{2}E_{2}\varepsilon_{t}\left(t\right)$$

where A and E are the cross-sectional area and elastic modulus, respectively. Knowledge of the forces on both sides of the specimen is important as this is the most widely used method of examining uniform loading in the specimen which is critical for a valid compression test. If the forces at each specimen side agree well, the specimen stress, *σ_s_*, can then be found as

(A11) $$\sigma_{s}\left(t\right)=\frac{F_{2}\left(t\right)}{A_{s}}.$$

Using only the transmitted bar force due to the reduced ringing in the bar signal. These calculations allow for the determination of the effect of the true strain rate on the true stress–strain behavior of materials.

Furthermore, in performing the quasi-static tests on the wet and dry brain, the following equations were used for calculating the true stress–strain response of the material. The engineering stress was given as

(A12) $$\sigma_{E}=\frac{P}{A_{0}}$$

where *P* is the force applied on the material, and *A_0_* the initial cross-sectional area of the sample. The engineering strain was defined as

(A13) $$\varepsilon_{E}=\frac{\ell -\ell_{0}}{\ell_{0}}$$

where *l_0_* is the initial height of the sample being tested, and *l* is the height of the sample at any given time. Using Equations (A12) and (A13), the true stress and strain were calculated using the following formulae:

(A14) $$\sigma_{T}=\;\sigma_{E}\left(1+\varepsilon_{E}\right)$$

(A15) $$\varepsilon_{T}=ln\left(1+\varepsilon_{E}\right)$$

Equations (A14) and (A15) were used for calculating the true stress–strain behavior of dry and wet brain samples at quasi-static rates.

## Appendix C

**Table A1:** Summary of symbol descriptions.

Symbol	Description
$U_{s}$	Wave velocity
$U_{p}$	Particle velocity
$c_{0}$, *s*	Wave velocity and particle velocity linear relationship constants
$\bar{\Psi }$	Free energy
$\dot{\bar{\psi }}$	Rate of change of the strain energy density function
${\bar{C}}^{e}$	Elastic part or the right Cauchy–Green tensor
${\bar{\xi }}_{1}$,${\bar{\xi }}_{2}$, ${\bar{E}}^{\bar{\beta }}$	Internal strain fields (internal state variables)
${\dot{\bar{\xi }}}^{*}$	Evolving strain threshold
${\bar{\xi }}_{\mathrm{sat}}^{*}$, ${\bar{\xi }}_{\;2\mathrm{sat}}$	Saturation value of ${\dot{\bar{\xi }}}^{*}$, ${\bar{\xi }}_{2}$
σ	Cauchy stress
**F**	Deformation gradient
$F^{e}$, $F^{eT}$	Elastic part of F, transpose of the elastic part of F
$F^{p}$ **,** $F^{pT}$	Plastic part of F, transpose of the plastic part of F
$J,\;J^{e},\;J^{p}$	Determinant of F, determinant of $F^{e}$, Determinant of $F^{p}$
${\bar{D}}^{e},\;{\bar{D}}^{p}$	Elastic rate of deformation, inelastic rate of deformation
$\bar{S}$	Second Piola–Kirchoff stress
${\bar{w}}^{p}$	Plastic rotational deformation
$R^{e},\;R^{eT}$	Elastic portion of the rotation tensor, transpose of the elastic portion of the rotation tensor
$U^{e}$	Right stretch tensor
$\bar{M}$	Mandel stress
$\tau_{1}$	Kirchoff stress (elasto-viscoplastic part)
μ	Elastic shear moduli
$\bar{I}$	Identity matrix
${\bar{E}}^{e}$	Elastic part of the Green–Lagrange strain
K	Bulk moduli
${\bar{\kappa }}_{1}$, ${\bar{\kappa }}_{2}$	Stress-like thermodynamic conjugates to ${\bar{\xi }}_{1}$,${\bar{\xi }}_{2}$
$\bar{\alpha }$	Stress-like thermodynamic conjugate to ${\bar{E}}^{\bar{\beta }}$
${\dot{\bar{\gamma }}}^{p}$	Viscous shear strain rate
${\dot{\bar{\gamma }}}_{0}^{p}$	Reference viscous shear strain rate
$\bar{\pi }$	Effective pressure
$\bar{\tau }$	Equivalent shear stress
αp	Stress-like thermodynamic conjugate of ${\bar{E}}^{\bar{\beta }}$
Y	Yield criterion
$\bar{\beta }$	Stretch tensor
$\dot{\bar{\beta }}$	Rate of stretch tensor
$h_{0}$, $g_{0}$, $h_{1}$	Hardening moduli
${\bar{B}}^{p}$	Intermediate plastic configuration
σ_t_	Elastic-viscoelastic transition stress
σ_p_	Elastic-plastic transition stress
${\bar{N}}^{p}$	Direction of viscous flow
$\dot{u_{1}}$	Hoppy bar velocities
t	Time
ε	Strain
$\dot{\varepsilon_{s}}$, $\varepsilon_{s}$	Hoppy bar sample strain rate, hoppy bar sample strain
$L_{s}$	Sample instantaneous length
$A_{1},{\;A}_{2}$	Hoppy bar areas
$F_{1},{\;F}_{2}$	Hoppy bar forces
$E_{1},\;E_{2}$	Hoppy bar elastic moduli
$\varepsilon_{i},\;\varepsilon_{t}$	Strain in the incident and transmitted hoppy bars
$\sigma_{s}$	Hoppy bar sample stress
$A_{s}$	Hoppy bar sample area
$\sigma_{E},\;\varepsilon_{E}$	Engineering stress, engineering strain
$A_{0}$	Initial sample area
**P**	Force applied on the material
$\ell ,\ell_{0}$	Current and reference lengths
$\sigma_{T},\;\varepsilon_{T}$	True stress, true strain
